# Fibroid Tumors of the Uterus

**Published:** 1899-04

**Authors:** Virgil O. Hardon

**Affiliations:** Atlanta, Ga.


ATLANTA

### Journal-Record of Medicine.

Successor to Atlanta Medical and Surgical Journal, Established 1855, and Southern Medical Record, Established 1870.

VOL. I. APRIL, 1899. No. 1.

BERNARD WOLFF, M.D., DUNBAR ROY, A.B., M.D., } EDITORS. M. B. HUTCHINS, M.D., BUSINESS MANAGER.

### ORIGINAL COMMUNICATIONS.

### FIBROID TUMORS OF THE UTERUS.*

BY VIRGIL O. HARDON, M.D.,

ATLANTA, GA.

Fibroid tumors differ from all other tumors in the fact that they are not, strictly speaking, pathological growth. They consist simply of an hypertrophy of normal tissue, being composed of the same elements that make up the structure of the uterine walls. They are made up of muscular tissue and of fibrous tissue in varying proportions. In some the fibrous tissue predominates, in others the muscular tissue, but in all fibroids both varieties of tissue are present, to a greater or less extent. They are not therefore, properly to be called fibromas, neither can they properly be called myomas, but the term fibromyoma, which is sometimes used, is the only correct one.

The hypertrophy of normal tissue which constitutes a fibroid tumor, differs from the usual forms of hypertrophy in the fact that it begins in a single bundle of muscular fibers, and grows by in-

* Remarks before the Atlanta Society of Medicine.

WI
AT4-59H

crease of that bundle in size, pushing before it the surrounding muscular fibers and thereby forming by pressure a surrounding capsule composed of muscular structure thickened by pressure. Hence, it is apparent that a fibroid tumor in itself is not a form of disease. Unlike abnormal growths, it has no tendency to invade surrounding structures, or surrounding organs, and does not involve any danger to life, or even any serious impairment of health, except in a mechanical way.

All fibroid tumors are histologically alike, hence the only classification of which they admit is one based upon their location with reference to the uterine walls. At the beginning, they are all intramural. As they increase in size, they grow in the direction of least resistance, which will always be toward that surface of the uterus which is the nearest to their point of origin. Hence, some, after increasing to such an extent that their diameters exceed the thickness of the uterine walls, project beyond the peritoneal surface, forming subperitoneal tumors, while others project upon the mucous side invading the uterine cavity and forming submucous tumors.

The contraction of the muscular tissue, by which a fibroid tumor is surrounded, has a constant tendency to force it out of its bed, thus increasing its projection beyond the surface of the uterus, and many are finally completely extruded, and remain attached to the uterus only by a pedicle, consisting of cellular tissue and the peritoneal or the mucous membrane which covers it. Such extruded tumors are known as polypi; subperitoneal, if attached to the outer surface of the womb, submucous if attached to the inner surface of the womb. A submucous polypus, lying in the uterine cavity, is still further subjected to the pressure exerted by uterine contraction, and may be forced out of the uterine cavity until it lies wholly within the vagina, remaining still attached to the inner surface of the womb by a long pedicle.

The causation of fibroids is obscure. They are for more frequent in the negro than in the white woman, probably from one third to one half of all the negro women past thirty years of age having fibroid tumors of great or less size. They are more common in unmarried, than in married women, and among married women, they are more common in those who have not borne children than in those who have. They occur most frequently in middle life. I have never seen one in a woman under twenty-five years of age. At the menopause the tendency to the formation of such tumors greatly diminishes and probably ceases altogether. Tumors which exist previous to the menopause frequently cease to grow, and even diminish in size after the change of life is established. This rule, however, is by no means invariable.

The symptoms produced by fibroid tumors are mechanical in their origin. They consist of hemorrhage and such symptoms as are caused by the pressure of the tumor upon the neighboring parts. Such pressure may produce pain or interfere with functions of other organs. Pressure upon the bladder, upon the rectum, and upon the intestines may all produce pain, but in some cases the effect of such pressure is limited simply to a disturbance of functions.

A tumor located wholly within the uterus is productive of far more hemorrhage than one growing from the fundus and projecting into the abdominal cavity. The hemorrhage is caused by the presence of the tumor as a foreign body within the uterine walls, or invading the uterine cavity. Small intramural tumors which do not project into the uterine cavity, as a rule, produce no hemorrhage. Subperitoneal tumors produce far less hemorrhage than submucous tumors. The subperitoneal polypus produces no hemorrhage, but the submucous polypus causes a greater flow of blood than any other variety of fibroid tumor. The submucous fibroid, and the intrauterine polypus have the same effect in this regard as would any foreign body within the uterine cavity, producing metrorrhagia and uterine contractions of a painful character. Thus it will be seen that the symptomatology of these tumors varies greatly with their location. The symptoms mentioned—pain, hemorrhage, and functional disturbance from pressure—are all that we find associated with fibroids. In many cases, especially of subperitoneal and intramural tumors, none of these symptoms are present, and these are the cases which frequently go on for years, or even for a lifetime, without calling for any treatment, and not infrequently, without the patient's knowledge of their existence unless they are discovered by accident.

The effect of fibroid tumors upon pregnancy deserves a moment's consideration. As has been already stated, these tumors occur most frequently in sterile women, but I have seen a number of cases where pregnancy occurred in spite of the presence of fibroid tumors of considerable size. Several years ago I was called to a patient who was four months advanced in pregnancy, and whose pelvis was also occupied by a subperitoneal fibroid polypus as large as a small cocoanut. Both the pregnant womb and the fibroid were incarcerated within the pelvis, and symptoms of threatened miscarriage were present. I opened the abdomen and removed the fibroid by tying and cutting off the pedicle, and the patient went on to full term of pregnancy. I saw a case about three years ago, with my friend Dr. Murphey, in which the woman was in labor, and on attempting to deliver her I discovered that she had five large fibroid tumors of the womb. The child was entangled, so to speak, among these fibroids, and the womb was unable to expel it. It was only after great exertion and considerable manipulation that the child was delivered. The mother made an uninterrupted recovery, the womb contracting firmly down in spite of the presence of the fibroids. In some cases, a fibroid tumor will prevent complete contraction after labor, and thereby cause post-partum hemorrhage. This is particularly the case when a large part of the uterine wall is occupied by a single tumor, thus rendering the womb unable to contract.

The treatment of fibroid tumors depends (1) upon the symptoms produced by them, and (2) upon their location. Tumors which produce no symptoms call for no treatment. If pressure symptoms be present, only in a moderate degree, they can frequently be relieved, at least temporarily, by lifting the womb in the pelvis and keeping it supported either by a pessary, or better still, by cotton or wool tampons, frequently renewed. If the pressure is so severe as to cause intolerable pain, or seriously interfere with the functions of neighboring organs, particularly the bladder and the bowels, then more radical measures must be resorted to. Hemorrhage may be either slight, a little every day, but in the aggregate, amounting to enough to seriously impair the patient's strength, or it may be profuse, occurring only at intervals, each attack of hemorrhage seriously depleting the patient and, perhaps, threatening her life. If the hemorrhage be moderate in amount and the tumor be intramural or subperitoneal, it can frequently be controlled by medication. As far as I know, there are only two drugs in the pharmacopeia which have the power of arresting uterine hemorrhage, and these are ergot and hydrastis. They act by lessening the amount of blood which goes to the womb. In many cases, hemorrhage caused by a fibroid can be effectually controlled by these drugs. But if the tumor be submucous, as a rule, such treatment will have but little effect. In such cases, the presence of the tumor in the uterine cavity as a foreign body is one factor in the production of the hemorrhage, while the irritating effect of the tumor within the uterine wall produces an engorgement of the whole pelvis, which acts as a second factor. This engorgement is shown to be present by the pulsation of the uterine artery, which can be detected by the finger in the vagina. In the normal condition of things, such pulsation cannot be felt. Hydrastis and ergot will lessen the engorgement, but they will not lessen the effect of the foreign body in the uterine cavity; hence, their failure to favorably affect the hemorrhage due to submucous tumors.

A thorough curetting of the uterine cavity will often prove effective in such cases. I remember a case which came to me in my clinic in the Atlanta Medical College, in which the patient suffered from profuse hemorrhage from a submucous fibroid. I curetted the womb thoroughly under an anesthetic, and there was no return of the hemorrhage for over a year. Dilatation of the cervical canal is said to have a favorable effect upon the hemorrhage. Theoretically, it is easy to see why this should be so. By virtue of the antagonistic action between the cervix and the body of the womb, whatever produces relaxation of the cervix produces a corresponding contraction of the body, and such contraction of the body, by lessening the amount of blood in the womb, has a tendency to control the hemorrhage. But in my experience, I have not found this theory to be carried out in practice. Slitting up the cervix bilaterally is also said to control the hemorrhage from fibroids, but I have found as little satisfaction in this method of treatment as in dilatation. When hydrastis and ergot fail, and when a thorough curetting of the uterus fails also, then nothing short of the removal of the tumor will control the hemorrhage. If the tumor be an intrauterine polypus, or a vaginal polypus, its removal is an exceedingly simple procedure. The tumor can be seized with a strong pair of forceps, drawn down as low as possible, the finger passed by it to locate the pedicle, and a pair of blunt-pointed, curved scissors, guided by the finger, can be passed up by the tumor, and the pedicle divided. The tumor is thus released, and is easily drawn out of the uterine or vaginal cavity. A subperitoneal polypus can be removed with almost as little difficulty, and almost as little danger. An incision through the abdominal wall can be made, the polypus drawn up through this incision, and its pedicle tied and divided with scissors or knife.

But when the tumor is located within the uterine wall, its removal is a much more serious matter. In such cases we have the choice of myomectomy, by which the tumor is shelled out of its bed, and the cavity closed by bringing its edges together with deep and superficial sutures, or hysterectomy, which involves the removal of all, or nearly all, of the uterus with the tumor.

Not all cases are suitable for the operation of myomectomy. When a considerable part of the uterus is involved, so that the removal of the tumor would leave a large opening between the uterine cavity and the abdominal cavity, which could with difficulty be securely closed, myomectomy is not practicable; and hysterectomy must be resorted to. Myomectomy is most applicable to subperitoneal fibroids, which do not involve the whole thickness of the uterine wall.

I shall not in this connection describe the technique of these procedures. It is sufficient to say that they are among the most formidable of abdominal operations and have a mortality considerably above that of the average of laparotomies. It is true that the improvement in the technique of these operations has notably lessened their danger in the past few years, and there is ground for reasonable hope that as time goes on their mortality will be still further diminished.

There is a certain class of cases in which the older operation of removal of the ovaries gives good results. These are the single tumors of moderate size, located in or near the fundus. In a considerable number of such cases the removal of the ovaries will control the hemorrhage, cause a diminution in the size of the tumor, and practically cure the patient. But in a minority, even of cases of this class, the results will be unsatisfactory, hence the operation is not a reliable one. In multiple tumors it usually fails altogether. For this reason the operation has practically passed into disuse, and the greater certainty of the operations of myomectomy and hysterectomy have caused them to be substituted for the removal of the ovaries.

